# A Web-Based Treatment Decision Support Tool for Patients With Advanced Knee Arthritis: Evaluation of User Interface and Content Design

**DOI:** 10.2196/humanfactors.8568

**Published:** 2018-04-30

**Authors:** Hua Zheng, Milagros C Rosal, Wenjun Li, Amy Borg, Wenyun Yang, David C Ayers, Patricia D Franklin

**Affiliations:** ^1^ Department of Orthopedics and Physical Rehabilitation University of Massachusetts Medical School Worcester, MA United States

**Keywords:** usability evaluation, patient decision support, knee osteoarthritis, total knee replacement, outcome prediction

## Abstract

**Background:**

Data-driven surgical decisions will ensure proper use and timing of surgical care. We developed a Web-based patient-centered treatment decision and assessment tool to guide treatment decisions among patients with advanced knee osteoarthritis who are considering total knee replacement surgery.

**Objective:**

The aim of this study was to examine user experience and acceptance of the Web-based treatment decision support tool among older adults.

**Methods:**

User-centered formative and summative evaluations were conducted for the tool. A sample of 28 patients who were considering total knee replacement participated in the study. Participants’ responses to the user interface design, the clarity of information, as well as usefulness, satisfaction, and acceptance of the tool were collected through qualitative (ie, individual patient interviews) and quantitative (ie, standardized Computer System Usability Questionnaire) methods.

**Results:**

Participants were older adults with a mean age of 63 (SD 11) years. Three-quarters of them had no technical questions using the tool. User interface design recommendations included larger fonts, bigger buttons, less colors, simpler navigation without extra “next page” click, less mouse movement, and clearer illustrations with simple graphs. Color-coded bar charts and outcome-specific graphs with positive action were easiest for them to understand the outcomes data. Questionnaire data revealed high satisfaction with the tool usefulness and interface quality, and also showed ease of use of the tool, regardless of age or educational status.

**Conclusions:**

We evaluated the usability of a patient-centered decision support tool designed for advanced knee arthritis patients to facilitate their knee osteoarthritis treatment decision making. The lessons learned can inform other decision support tools to improve interface and content design for older patients’ use.

## Introduction

Arthritis, with its most common form osteoarthritis (OA), affects 50% of all adults older than 65 years of age and is the most common chronic condition and cause of disability in the United States [1]. When knee OA pain and disability advances, total knee replacement (TKR) surgery can effectively eliminate pain and improve function. Total knee replacement is now the number one most common procedure among hospital discharges [2]. Knowledge about the medical and surgical treatments and associated outcomes is critical for patient decision making. Patients who delay the procedure until late in the symptom course may have less optimal results [3].

Hudak and team [4] evaluated the reasons that prevented some elderly OA patients from considering total joint surgery. Ongoing deliberation of the surgical option mainly resulted in a deferral of the treatment decision. The barriers to limiting surgical decision making include inaccurate estimation of symptom level for surgical candidates and lack of information to discuss with their physicians. Our recent review confirms the lack of published research on shared decision making and patient decision aids in orthopedic surgery, with no evidence about the use of patient decision support tools for knee OA patients considering TKR [5]. Therefore, patient decision support tools for the advanced knee arthritis population are needed to help them understand their individual OA symptom severity, provide evidence-based benefits and risks, and aid communication with their physicians to guide treatment decisions.

The average knee OA patient who chooses surgery is 66 years of age [2], and the user interface both for data entry and data output must be designed to facilitate ease of use among aging adults and minimize potential barriers. The objective of this study was to examine user experience and acceptance of a Web-based treatment decision support tool for advanced knee arthritis patients who are considering TKR surgery. Our results may inform user interface and outcome presentation design for other decision support tools for older adults with diverse health conditions.

## Methods

### Tool Development

The tool’s user interface was designed by a multidisciplinary team including an orthopedic specialist, a researcher with expertise in health literacy, a computer scientist, and a biostatistician. The team focused on developing a user interface design that would be simple to operate by older adults with functional limitations such as vision decline and diminished motor skills. To facilitate use among low literacy individuals, the tool used white background and dark text, one-question-per-page display, big font and simple layout, and plain language within eighth-grade literacy reading level.

Briefly, the tool prompts patients to respond to 20 questions related to demographics, overall health, knee pain and function, medical comorbidities, and expectations one year after surgery. Using data entered by the patient, the tool estimates likely individual patient-level improvement in post-TKR pain relief and physical function according to patient characteristics and current health attributes. These estimates are then translated into metrics meaningful to patients (ie, pain relief at rest, pain relief when walking, and ability to walk five blocks at a year after surgery). These metrics are easily understood by patients and can be used to facilitate communication between patients and surgeons and thus support TKR decision making.

### Patient Recruitment

The study sample was recruited from the UMass Memorial Health Care Arthritis and Total Joint Center. All patients aged 21 years of age and older seeking knee OA care at the Arthritis and Total Joint Center were eligible. Patients with acute knee injuries or who were not fluent in English were excluded.

A study recruiter screened all new pre-TKR and post-TKR patients during the study months. After confirming eligibility, a study coordinator contacted each potential participant by telephone to describe the study and invite him/her to participate. If the patient was willing to take part in the study, the study coordinator scheduled an interview before or after the next doctor’s appointment, and mailed a fact sheet, a consent form, and a HIPAA authorization form to the patient for signature. At the interview, the study coordinator answered any questions and gave a copy of the consent form to the patient in case he/she did not bring the signed one. Patient participants received a stipend of US $10 for parking at the end of the interview. The study was approved by Institutional Review Board for the protection of human subjects.

### Usability Evaluation Procedures

User-centered formative and summative evaluations were employed for the tool usability testing [6]. The first phase goal, the formative evaluation, was to improve the tool design through participants’ response to preliminary ideas of design. This was accomplished through a first round of evaluation interviews. The second phase, designed as the summative evaluation, was to assess the clarity of outcome information as well as usefulness, satisfaction, and acceptance of the tool through interviews and questionnaires. This was accomplished through a second round of evaluation interviews. Methods used in each round are described subsequently.

#### Round 1

Round 1 was performed based on the iterative evaluation process; the tool was adjusted after each subround of interviews and was then reassessed in the next subround. To avoid bias, different participants were recruited in each subround. Round 1 interviews started with a survey of patient demographics and computer abilities. Participants were asked to use the Web-based tool on the computer and encouraged to think aloud their immediate feelings as they completed each survey page and task. The think-aloud method was used to verbalize users’ thoughts, feelings, and opinions while interacting with the system. Thinking aloud slows the thought process and increases mindfulness, which is very helpful for capturing a wide range of cognitive activities. During the use of the tool, the participants were asked questions about tool design. The questions were structured with predetermined topics, such as wording, layout, color, button and overall utility, as well as with open-ended comments. The overall duration of the interview was up to 30 minutes. The process was administered by a study coordinator with expertise in patient interviews.

#### Round 2

Round 2 was a summative evaluation to conduct an overall assessment of the near-final version of the tool. Round 2 patients were asked to report their demographics and computer abilities at the beginning of the interview. They then completed the survey questions of the tool with no interruption, followed by an interview about their opinions about the presentation of outcome information. Five types of presentations were provided: text summaries, bar graphs, word clouds, smiling faces, and staged walking people. Interview items assessed the format that was liked best / liked least, the ease of understanding, and the helpfulness for decision making. Finally, participants completed a standard Computer System Usability Questionnaire (CSUQ) [7], a standardized assessment to measure user satisfaction with computer system usability and four customized research questions on technical difficulty and tool format preference (Web vs paper). The interview process took up to 30 minutes. The number of participants enrolled in round 2 was based on group size-specified guides for quantitative usability studies [8,9].

### Data Collection Tools

Patient participants completed study procedures in a quiet room adjacent to the Arthritis and Total Joint Center. A desktop computer allowed access to the Web-based tool with survey questions and outcome data display. Screen recorder software, Camtasia Studio 6, was used to captured user’s operations on the computer screen, such as cursor movement, mouse clicking, and keyboard input. A digital voice recorder taped the comments and discussion during the process. Participants’ gender, age, education level, and computer use were asked on a one-page demographics and computer ability survey. An interview guide was developed by the study team based on user-centered formative and summative evaluations. A trained interviewer administered patient interview process and a usability specialist acted as primary observer.

### Data Analysis

Patient demographics and computer ability data were analyzed descriptively. Means and proportions were used to describe the characteristics of the study sample. Qualitative analysis summarized findings from the interview and observation data into several topics, enumerated the patients’ needs and preference of design of the tool. Quantitative data included time spent on each page and in total on the use of the tool and usability scores which assessed usefulness, satisfaction, and acceptance of the tool.

## Results

### Participant Characteristics

For round 1, 11 patients were contacted and 8 (73%) participated in the study. For round 2, 20 patients were contacted and all (100%) participated. Participant characteristics are shown in Table 1. For all 28 patients, the mean age was 63 (SD 11) years and 12 (43%) were older than 65 years; 21 (75%) were female, 26 (93%) had at least a high school education level, 15 (54%) used a computer every day, and 9 (32%) rarely or never used a computer. Eight of nine participants with low computer use were 65 years of age or older.

### Round 1 Findings

Eight participants were involved in round 1 and three subrounds were conducted during the iterative design process. The findings are categorized by information clarity and interface design tasks.

#### Information Clarity

Most participants had no difficulty in understanding the survey questions. They felt that the language was simple and the wording was easy to understand. The only unclear item was the use of the word “knee scope” in a question about prior surgery. After we changed “scope” to “arthroscopic surgery (a scope inserted by a doctor into your knee),” participants agreed that the presentation was clear. Some participants suggested asking questions for knee pain and physical activity on a good day, a moderate day, and a bad day.

**Table 1:** Participant characteristics (N=28).

Patient factors		Round 1, n (%) (n=8)	Round 2, n (%) (n=20)
**Gender**			
	Female	7 (88)	14 (70)
	Male	1 (12)	6 (30)
**Age (years)**			
	<65	6 (75)	10 (50)
	≥65	2 (25)	10 (50)
**Education**			
	Less than high school	0 (0)	2 (10)
	Attended or graduated from high school/GED	3 (37)	7 (35)
	Attended or graduated from college	5 (63)	11 (55)
**Computer use**			
	Every day	5 (63)	10 (50)
	Once a week	0 (0)	3 (15)
	Less than once a week but more than once a month	0 (0)	1 (5)
	Rarely or never	3 (37)	6 (30)

**Figure 1:**
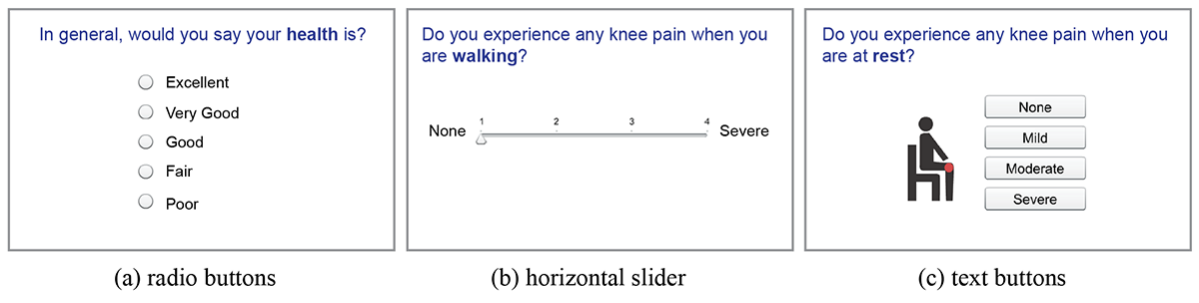
Questionnaire recording patients’ buttons preferences.

#### Interface Design

##### Layout

The questions were organized as one per screen, which was reported as clear and easy to read. Arial was used as the main font and all participants liked it. Font size was modified from 15-18 points to 18-20 points because 15 points was too small. Patients reported that some screens looked similar; for example, knee pain when walking and at rest. Based on this input, we modified the screens to include relevant images, such as someone walking or sitting, and highlighted important words in bold to clarify the difference in question focus.

##### Buttons

Radio buttons, horizontal sliders, and text buttons (Figure 1) were tested by participants to indicate their answer to a Likert-scale question. Many participants suggested using bigger radio buttons to click. Most participants, especially those who use a computer infrequently, advised against slider buttons because they found that “clicking and dragging” is hard to operate. Text buttons were thought better and easier to click. A “Next Question” button was initially put on each screen with a “Previous Question” button. Some participants expressed the need for a reminder to click on the “Next Question” button. Therefore, we used “automatic jump” to next screen by selecting an answer instead of an extra click on a “Next Question” button. Participants preferred this automatic function.

##### Colors

We used dark text on a white background for tool screens. No patient had problems with this style. One participant with glaucoma said questions were easy to read. The topic of each question was highlighted on the top of the screen with white text on a dark background; colored backgrounds were initially used to represent different categories of topics; for example, orange for demographics, blue for knee condition, but some participants did not like the colors. To simplify, the final version only used blue for topic background. One participant suggested color-coding the answer to a question in red, yellow, or green when it is relevant, such as red for severe pain and green for no pain, to highlight different selections.

##### Images

We added images to some of the questions for better comprehension. Most of participants reported that images made questions visually distinct. Numerous participants preferred images with a real person as compared to a “fake” person and one participant did not like cartoon images. A computer-savvy participant felt little attention was given to images compared to words.

### Round 2 Findings

Based on the problems identified in round 1 usability testing, we revised the design of the tool. Twenty patients participated in round 2 and tested the enhanced version. Round 2 focused on the testing of the presentation of the outcomes and usefulness, satisfaction, and acceptance of the tool.

#### Preferred Outcome Presentations

Five outcome presentation formats were shown to participants: text summaries, bar graphs, word clouds, smiling faces, and staged walking people (Multimedia Appendix 1). Participants could choose up to two preferred presentation formats. They easily distinguished which result format appealed to them more, and had clear reactions to different presentations. Bar graphs and staged walking people were preferred overall.

#### Time Spent on Tool and Each Screen

**Figure 2:**
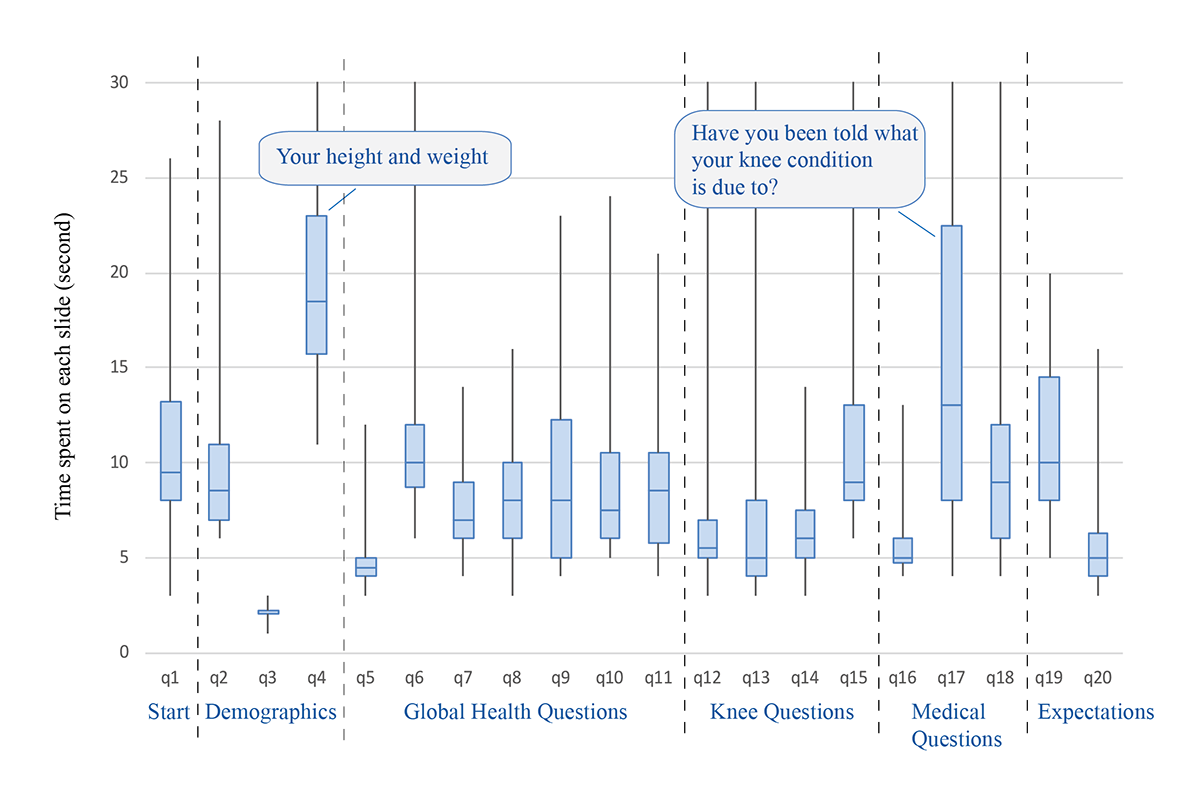
Distribution of the time spent on each page of the tool.

A total of 19 of 20 Camtasia data records from round 2 were captured; one record was not saved due to an operational error. Four participants seemed unfamiliar with computer use from their records of mouse clicking and keyboard entry. Two of them were older patients who were not able to use the computer themselves and asked the interviewer to operate the mouse for them. Considering the remaining 15 participants, the total time spent on the tool varied between 2 and 4 minutes, and the mean time spent on each screen was 9.7 (SD 4.2) seconds (Figure 2).

**Figure 3:**
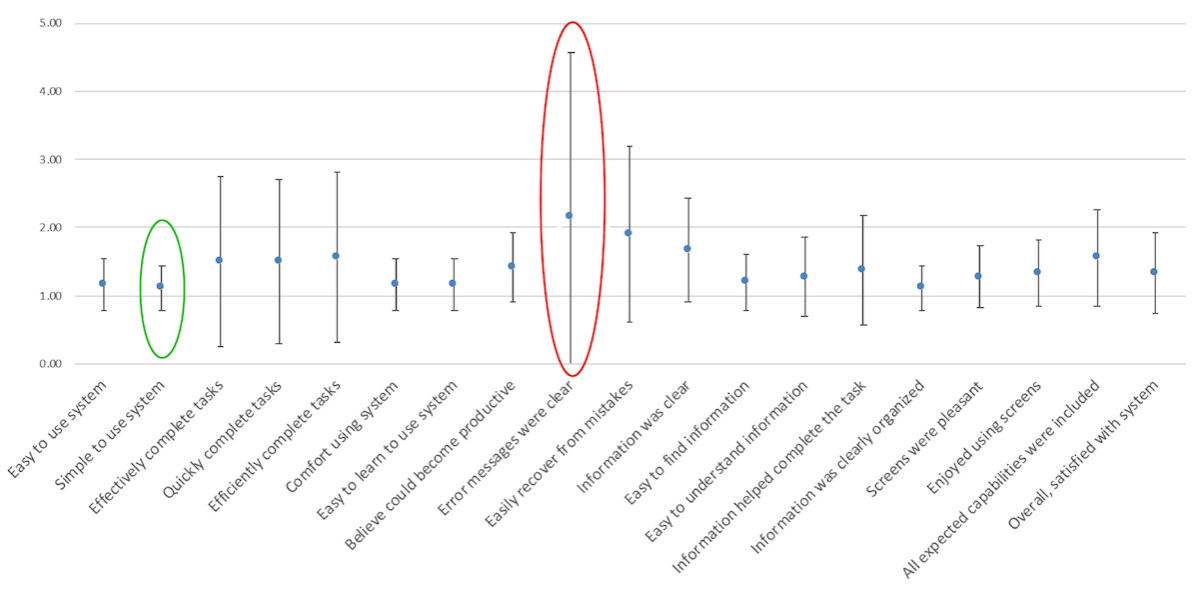
Computer System Usability Questionnaire item scores.

Two questions took participants a longer time than others to answer: (1) What is your height and weight? (to answer this question, a participant had to move the cursor to three different boxes and type in their answers), and (2) Have you been told by a health care provider what your knee condition is due to (one of the following)? The distribution of the time spent on each question is in Figure 3.

#### User Satisfaction Scores

A total of 19 of 20 participants in round 2 completed the usability evaluation survey. The questionnaire included 19 items with a Likert scale ranging from 1 (“strongly agree”) to 7 (“strongly disagree”) to measure user satisfaction. Low scores are better than high scores. The mean CSUQ scores on the four items about system usefulness, information quality, interface quality, and overall were 1.32 (SD 0.55), 1.44 (SD 0.58), 1.39 (SD 0.37), and 1.37 (SD 0.41), respectively. The mean and SD for each item can be found in Figure 3. Satisfaction was greatest for simplicity of use (mean 1.11, SD 0.32) and lowest for error message (mean 2.17, SD 2.40). The responses to four customized research questions revealed that 74% (14/19) of the participants had no technical questions using the tool, and 84% (16/19) could use the tool without instructions. In all, 68% (13/19) of the participants preferred a computer version compared with a paper version, and 26% (5/19) thought either was fine. Finally, 89% (17/19) of participants reported that they would recommend this tool to a friend.

## Discussion

### Role of Web-Based Treatment Decision Tools

Computerized decision support tools are a new approach to treatment planning [10]. In contrast to patient education systems, decision tools provide information about a recommended treatment plan, including potential benefits and harms, and provide a foundation for patients to make a data-driven decision between two or more treatment options. Web-based interactive tools can facilitate this process by accessing online health information and helping patients get informed treatment options before communicating with doctors [11-13].

### Lessons Learned on User Interface

Through usability evaluation of a Web-based patient-centered decision support tool for advanced knee OA patients, we learned the preferences of older OA patients to inform tool design. Textbox 1 is the summary of patient preferences.

Aging adults are an important and understudied group for evaluation of Web survey usability and outcome data presentation. Their needs and concerns may differ from those of other age groups due to the natural changes associated with the aging process. The literature on Web accessibility for older users describes aging-related functional limitations, such as vision decline, motor skill diminishment, and cognitive decline [14,15].

The guidelines for accessible content include large print, simple language, and easy navigation. Our findings are consistent with prior research. For example, the participants liked larger fonts, larger text-filled buttons, fewer colors, simpler navigation without extra “next page” click, less mouse movement, and clear illustrations with simple graphs. Advanced functionality can cause usability difficulties for older adults. For example, horizontal sliders are a common element in Web design, but none of the participants liked them and they reported “am not able to manipulate” or “have difficulty figuring out how to do.”

**Summary of patient preferences.**

**Interface**

***Text***
  1. Sans serif font, such as Arial
  2. Big font size of 18 points or more
  3. Highlighting important words
***Buttons***
  1. Big text buttons; no slider bars
  2. Automatic jump to next page by selecting an answer instead of an extra click on “Next” button
  3. Avoid operations that need more mouse movement
***Colours***
  1. White background and dark text
  2. Fewer unnecessary colors
***Images***
  1. Simple images for illustration
  2. Eliminating distracting images

**Information**

***Clarity***
  1. Plain language instead of medical terms
  2. Short description for necessary medical terms

**Predictive Outcome Measures**

***Preferences***
  1. Clear and easy to understand, such as bar charts
  2. Outcome-specific with positive action, such as walking people for arthritis patients

Medical terminology is usually a significant obstacle for patients. Past research has revealed that participants experience difficulties understanding jargon, especially medical terminology [14]. The team’s health literacy specialist advised us to avoid medical jargon and improve explanations during the tool development. Thus, most participants reported no problems understanding the information presented. For medical terms that are difficult to simplify, such as arthroscopic surgery, we used both the term and a short description (ie, a scope inserted by a doctor) and learned that this way effectively conveyed the medical information.

The results also revealed that an easy-to-use system is more important than a comprehensive user manual. Most of the participants preferred the computer version over a paper survey. The most recent Pew reports released in 2018 showed that 66% of American adults ages 65 and older use the internet, and 73% of people aged 50 to 64 years and almost one-half of people aged 65 and older own a smartphone [16,17]. Most aging baby boomers will use computerized and mobile tools in the future, so we anticipate growing ease of use.

### Lessons Learned on Presenting Outcomes

Presenting likely outcomes of surgical procedures can provide new insights to patients about possible benefits and risks. Tailored estimates of the likely benefits of TKR surgery based on specific patient profiles are feasible using current computing technologies. However, the manner of presentation of predicted outcomes affects how patients understand the value of a treatment and may influence patients’ decisions [18,19]. For example, among key TKR outcome research publications, outcomes were expressed as global health assessment scores such as the Short Form Health Survey, the Veterans RAND 12-item Health Survey, or Patient-Reported Outcomes Measurement Information System Global Health survey, or knee-specific pain and function scores such as the the Western Ontario and McMaster Universities Osteoarthritis Index or Knee injury and Osteoarthritis Outcome Score survey. Global and knee outcome metrics are useful to clinicians and researchers, but do not convey to patients likely achievable and meaningful outcomes. In this study, we translated outcome measures into meaningful metrics to patients, such as pain-free walking, or home and community activity levels. The metrics were easily understood by patients, which can facilitate informed communications between patients and surgeons.

In addition, outcome data can be illustrated in different ways and patient comprehension may differ when information is presented using different words or displays to communicate [20-22]. To explore patients’ preferences on presentation of outcome data, we evaluated five different presentation formats. It was hypothesized that older adults might prefer text more than numbers, but only a few people chose text display. Color-coded bar charts made more sense to them and were reported to be “clearer” and “easy to understand.” These findings are consistent with prior studies that found that bar charts were most commonly preferred and least often found difficult to interpret [20,23]. We were surprised that participants liked the staged walking people display, a combination of graphs and numbers. Walking people graphs were thought more user-friendly and easy to understand, and suggestive of their primary goal—greater activity. We learned that older adults understand and accept outcome-specific graphs with positive action to present data.

### Study Limitations

Study limitations include a relatively small sample. However, user interface evaluation research has reported that 31% of usability problems can be identified with a single user [24], and more than 80% of usability problems can be identified with a sample of five users [25,26]. Thus, it is likely that the size of our sample was sufficient to identify most interface design problems. Culturally and linguistically diverse patients were not considered in patient selection in this study. We plan to test the tool in a broad and diverse national sample in the future.

### Conclusion

We evaluated the usability of a patient-centered decision support tool designed for advanced knee arthritis patients to facilitate their surgical treatment decision making. Patient participants showed high satisfaction and acceptance of the usefulness and interface quality of this easy, simple tool and selected acceptable data presentation formats for understanding of predictive outcomes after surgery. We expect to collect more data in future studies to verify the qualitative and quantitative findings. Our experience with the tool user interface and outcome presentation design for knee OA patients can inform the design for other chronic conditions within elderly populations.

## Appendix

Multimedia Appendix 1 Votes for outcome presentations.

## Abbreviations

CSUQ: Computer System Usability Questionnaire
OA: osteoarthritis
TKR: total knee replacement
