# Supplementary material for: A Web-Based Treatment Decision Support Tool for Patients With Advanced Knee Arthritis: Evaluation of User Interface and Content Design
Source: JMIR Hum Factors. 2018 Apr 30;5(2):e17. doi: 10.2196/humanfactors.8568 (PMC5952113; doi:10.2196/humanfactors.8568)
Supplement: Multimedia Appendix 1 [file humanfactors_v5i2e17_app1.pdf]

Votes for outcome presentations.

<table>
<tr>
<th></th>
<th>Text
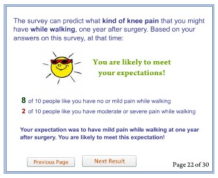
</th>
<th>Bar graph
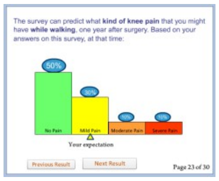
</th>
<th>Word clouds
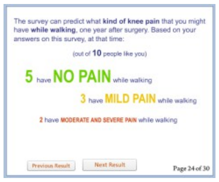
</th>
<th>Smiling faces
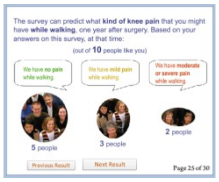
</th>
<th>Walking people
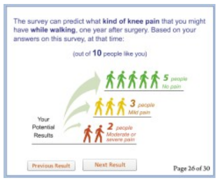
</th>
</tr>
<tr>
<td>Number preferring</td>
<td>3</td>
<td>10</td>
<td>2</td>
<td>1</td>
<td>9</td>
</tr>
<tr>
<td>Reasons for preferring</td>
<td>-Positive
-Likes text words
-Easy to understand</td>
<td>-Clearer
-Easy to understand
-Likes numerical or percentage</td>
<td>-Clear</td>
<td>-Likes real people telling expectations</td>
<td>-Shows action
-More information, more friendly
-Easy to understand</td>
</tr>
</table>
